# Effects of Crosslinking and Silicone Coupling Agent on Properties of EVA Composite Hot Melt Adhesive

**DOI:** 10.3390/polym13234101

**Published:** 2021-11-25

**Authors:** Zijin Wu, Yonggang Shangguan, Chunhui Zhang, Qiang Zheng

**Affiliations:** 1 MOE Key Laboratory of Macromolecular Synthesis and Functionalization, Department of Polymer Science and Engineering, Zhejiang University, Hangzhou 310027, China; 21829026@zju.edu.cn; 2 Zhejiang Liniz New Material Co., Ltd., Hangzhou 311300, China; z_chunhui@sina.com

**Keywords:** EVA, hot melt adhesive, crosslinking, silicone coupling agent

## Abstract

In order to improve the bonding performance, EVA composite hot melt adhesives were prepared by introducing crosslinking agent and silane coupling agent in this paper. A binary EVA resin blend as the base resin with appropriate viscosity and tensile shear strength was selected as hot melt adhesive. The effects of crosslinking agent and silane coupling agent on the properties of ethylene/vinyl acetate (EVA) composite hot melt adhesive were studied. By investigating the preparation and curing conditions of hot melt adhesive and the properties of hot melt adhesive after the introduction of dicumyl peroxide (DCP), the optimum temperature and dosage of DCP and its influence on the properties were determined. It was found that the tensile shear strength of hot melt adhesive increased from 0.247 MPa to 0.726 MPa when 2 phr DCP and 5 phr KH570 were added at the same time. The tensile strength and tensile shear strength of hot melt adhesive are only slightly improved when silicone coupling agents with different functional groups are added to EVA composite hot melt adhesive. However, it was found that excessive silane coupling agent would significantly reduce the tensile strength and shear peel strength of the material.

## 1. Introduction

Hot melt adhesive is a plastic, solvent-free adhesive. It is a solid at room temperature and melts into viscous liquid after being heated to a certain temperature. Since hot melt adhesive has many advantages, such as being non-toxic and environmentally-friendly, fast bonding, convenient to transport and store, and with a wide range of applications, recently hot melt adhesives have been widely used, from traditional packaging fields to clothing, shoemaking and automobiles etc. [1,2,3,4,5]. The commonly commercial hot melt adhesives are mainly divided into the following categories: polyurethane (PU), polyamide (PA), EVA and their derivatives or modifications. Among them, EVA and its derivatives are one of the most used. Since EVA is a copolymer of ethylene and vinyl acetate (VA), its crystallinity becomes relatively small depending on the VA content. Therefore, EVA presents better flexibility, elasticity, and impact resistance than polyethylene. Taking into account the fact that the primary function of EVA in hot melt adhesives is to produce the necessary bonding strength and cohesive strength, EVA used as hot melt adhesives should have the following properties: good thermal stability, a wide range of applicability, high bonding strength and good compatibility with other components [1]. Previous studies have shown that the VA content of the EVA resin for preparing hot melt adhesives is generally between 6 and 40% [2]. As the VA content gradually increases, the flexibility and viscosity of the EVA resin will increase and the bonding strength will increase, but the fluidity, hardness, and melting point will decrease accordingly. Therefore, EVA resin with appropriate VA content should be selected as the main component of the hot melt adhesive according to the requirements for the properties and application process of hot melt adhesive. However, the low bonding strength and low temperature resistance of EVA resins make it impossible to adapt to the requirements of industrial development by using EVA alone, and thus graft modification [6], blending modification of EVA [7,8,9,10,11] and reactive hot melt adhesives [12,13,14] are necessary for the preparation of high-performance EVA hot melt adhesive.

The primary component of EVA hot melt adhesive is usually linear ethylene-vinyl acetate copolymer, and consequently, it has poor heat resistance and cohesive strength. To improve these shortcomings, it can be used with several modifiers, such as crosslinking agent and coupling agent [12,13,14]. For the former case, EVA hot melt adhesive undergoes a crosslinking reaction upon heating to form a three-dimensional network structure, which can significantly improve the heat resistance, cohesive strength, bonding strength, solvent-resistance and other properties of hot melt adhesive [15]. Zhao et al. [12] used different crosslinking systems to probe the influence of EVA content in the glue and the VA content in EVA component on the degree of crosslinking. Wei et al. [13] presented novel performances of shape-memory materials made by crosslinking reaction between polymers and crosslinking agents. Xu et al. [14] crosslinked EVA by adding DCP and elevating temperature, and they found that the degree of crosslinking can be influenced by the amount of the peroxide and the time and temperature of the reaction. The organic silane coupling agent can also be used as a tackifier for adhesives and sealants; it can be used as a primer for substrates, or can be directly added to the material by blending to enhance the bonding strength of the adhesive. Su et al. [16] added vinyl tert-butyl silane (VTPS) into silicone rubber and fluorosilicone rubber, and found that VTPS was a good tackifier for high-temperature vulcanization of silicone rubber. The addition of VTPS can remarkably increase the bonding strength between silicone rubber and aluminum alloy or stainless steel. Zhang et al. [17] formulated various adhesives containing silane coupling agents, and performed surface treatment on the fabrics. They found that, although the mass fraction of the coupling agent in the adhesive only accounts for 3%, it has a great influence on the bonding strength. Regarding the mechanism of organosilane coupling agents, some theories [18], including chemical bond theory [19], surface infiltration theory [20], deformation layer and constrained layer theory [21] and reversible hydrolysis bond mechanism [22] etc. have been proposed. These theories have discussed the role of silicone coupling agent in the system from a certain viewpoint and can partially explain its interpretation of the experimental results, but so far, there is no unified theory [18]. In this study, EVA compound was prepared by introducing silane coupling agent and initiating by peroxide to improve bonding strength and low temperature resistance of hot melt adhesive. The influence of the amount of peroxide and the crosslinking temperature on the adhesion and tensile properties of EVA resin were explored [12,13,14,15,16,17]. In addition, the modification effects of different organic silane coupling agents were investigated.

## 2. Experimental

### 2.1. Materials

Four EVA copolymers were used in this work. The three Elvax EVA copolymers were supplied by DuPont Corp (Wilmington, DE, USA). and the EVA43 was supplied by Macklin Biochemical Co. Ltd. (Shanghai, China). Some characteristics of EVA copolymers, such as the vinyl acetate contents, melt index (MI) and molecular weights, are shown in Table 1. Three Elvax EVA copolymers were chosen for their high melt index, which is good for melt processing, and their appropriate VA content is suitable for making hot melt adhesives. EVA43 was selected to adjust the bonding strength of EVA resin.

Three kinds of silane coupling agent supplied by Macklin Biochemical Co. Ltd. (Shanghai, China) with different functional groups were used in this work and their structure are shown in Table 2. These three kinds of silane coupling agent were chosen because they all have polar groups in addition to the -Si-O- groups, which is beneficial to enhance the polarity of the hot melt adhesive. The DCP used here was supplied by Aladdin Corp. (Beijing, China).

### 2.2. Sample Preparation

To prepare the EVA compound hot melt adhesive, various components are weighed as the designed mass ratio and blended in a 500 mL stirred reactor provided by Ketu Machinery Technology (Shanghai, China) and stirred at 60 rpm for 45 min at 100 °C, which ensures that EVA can melt and has certain fluidity so that DCP can be dispersed evenly, while also avoiding reaction caused by DCP decomposition. Then, after the resulting compounds were hot-pressed on a flat vulcanizer provided by Xingli Corp.(Huzhou, China) for 10 min at 100 °C, it was cooled down to room temperature. The purpose of the latter step is to remove possible residual bubbles in the compounds so as not to affect the bonding effect of the adhesive.

### 2.3. Characterization

#### 2.3.1. Gel Permission Chromatography (GPC)

The molecular weights and molecular weight distributions of the EVA copolymers were measured using a gel permeation chromatography (GPC HT-806M, Shodex linear column, Waters, Milford, MA, USA). EVA copolymer was completely dissolved in tetrahydrofuran (THF) provided by Macklin Biochemical Co. Ltd. (Shanghai, China) as solvent with the concentration of 1 to 5‰ in weight.

#### 2.3.2. Differential Scanning Calorimeter (DSC)

The melting temperature of EVA copolymers and their compounds were measured using a differential scanning calorimetry (DSC25, TA Instrument, NewCastle, DE, USA) at a heating rate of 5 °C/min. The scanning circles consist of cooling from room temperature to −90 °C and subsequent heating to 150 °C again at a rate of 5 °C/min.

#### 2.3.3. Tensile Strength and Fracture Length Ratio

Tensile strength and elongation at break were measured using a stretching machine provided by Suns Technology Stock Co. Ltd. (Shenzhen, China). The crosslinked EVA compound samples were cut into a dumbbell shape with a width of 4mm and a thickness of 1.7 mm. A load to 5 kN and a tensile rate of 500 mm/min was applied to tensile tests until breaking, and the tensile stress at break and the elongation of the sample were recorded. The result was an average of five specimens.

#### 2.3.4. Tensile Shear Strength

Polycarbonate (PC) sheets provided by Leshi Corp. (Shanghai, China) were used to test the tensile shear strength of the substrates adhered by EVA compound hot melt adhesive. After EVA compound hot melt adhesive was melted at 140 °C for 5 min, it was applied to the surface of the substrate to complete the bonding and then the bonded specimen was stored in an oven at 45 °C for 24 h. Tensile shear strength was measured using a stretching machine provided by Suns Technology Stock Co. Ltd. (Shenzhen, China). A load of 5 kN and a tensile rate of 5 mm/min were applied to tensile tests until breaking. The bonding surface and the clamping surface of the fixture are as shown in the Figure S1 below in Supporting information.

#### 2.3.5. Swelling Test

Take the vulcanized blends with a mass of *m*_0_ in a certain amount of THF, soak it at room temperature, change the solvent every 12 h, take it out after the mass of the swollen sample is measured stable, and weigh the final swollen mass as *m*_1_. Dry the sample fully in an oven, and the mass of the sample after drying the solvent is *m*_2_. The gel content in the sample is *m*_2_/*m*_0_. The swelling degree *Q* is calculated in the following formula:

$$Q=\frac{\frac{m_{1\;}-m_{2}}{\rho_{s}}+\frac{m_{2}}{\rho_{r}}}{\frac{m_{2}}{\rho_{r}}}$$

where *ρ_s_* and *ρ_r_* indicate the density of solvent and the blends, respectively.

## 3. Results and Discussion

### 3.1. Tensile Shear Strength of EVA Resin

The tensile shear strength of three EVA copolymers produced by DuPont on PC sheets and their melting temperature were tested. As shown in Table 1, Elvax220w has the strongest tensile shear strength among them. This is due to the fact that Elvax220w has a higher VA content or a lower melt index than the other two, therefore Elvax220w was chosen as the main substrate of hot melt adhesive.

**Figure 1:**
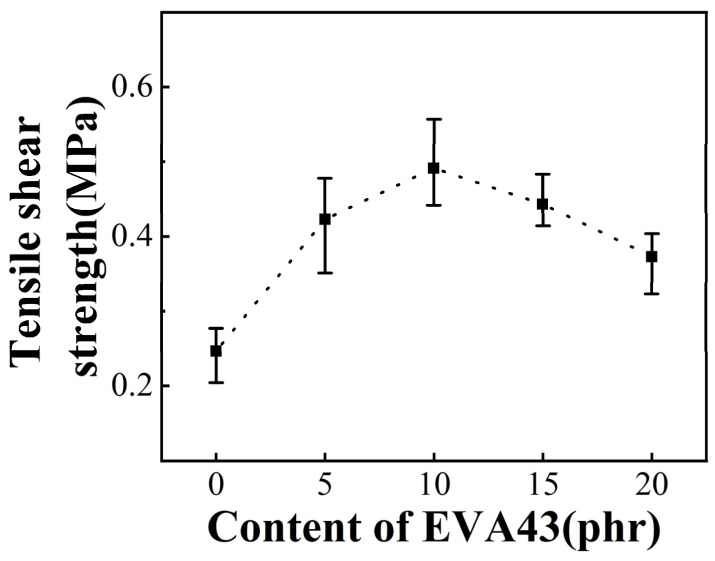
Tensile shear strength of Elvax220w/EVA43 blends with different composition.

Because the tensile shear strength of Elvax220w itself is not high, a certain amount of EVA43 resin with higher viscosity which presents similar melting behavior as shown in Figure S2 was added to improve adhesion strength. Figure 1 shows tensile shear strength of Elvax220w/EVA43 blends with different composition. As the content of EVA43 increases, the tensile shear strength of the blend sample increases first and then decreases, reaching the maximum when the content of EVA43 is 10 phr. The viscosity of EVA43 is higher than that of Elvax220w, so a small amount of EVA43 can effectively increase the tensile shear strength of blend sample. However, when the content of EVA43 continues to increase, the viscosity of the blend sample increases and its fluidity significantly reduces. As a result, the wettability between the blend sample and PC sheets will decline, resulting in a reduction in tensile shear strength.

### 3.2. Influence of DCP

As mentioned in the Introduction section, appropriate crosslinking can improve the mechanical properties of EVA resin. In this work, DCP was selected to initiate the crosslinking of EVA to increase the strength of the EVA resin itself and the bonding strength between adhesive and PC sheets. At the same time, it is necessary to ensure that the EVA resin has good flexibility in the preparation process.

**Figure 2:**
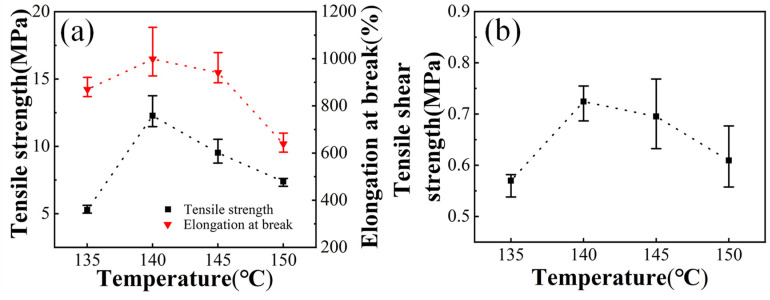
Tensile strength, elongation at break (**a**) and tensile shear strength (**b**) of Elvax220w samples with 2 phr DCP at different crosslink temperature.

In order to ensure that the sizing process can be completed in a relatively short time and to prevent the crosslinking speed from being too fast, thus causing the fluidity to drop too fast to complete the sizing, an appropriate crosslinking temperature of DCP should be first determined according to the half-life time at different temperatures of DCP, as shown in Table S1 in the Supporting Information. Figure 2 gives the tensile strength, elongation at break and tensile shear strength of Elvax220w resin at different temperature, respectively. Here, 2 phr DCP was used. It can be seen that with the increase of temperature, the tensile strength, elongation at break and tensile shear strength of the sample first increase and then begin to decrease continuously. All of them reach the maximum at 140 °C. These results may be due to the chemical bonds formed by crosslinking as the temperature increases and the degree of crosslinking gradually increases. When the degree of crosslinking further increases, the formed crosslinked network gets tighter, and the originally slack polymer chain is stretched, which made it easier to be broken. In addition, the tensile shear strength of crosslinked EVA samples also presents similar trend, which may be ascribed to the increase in the degree of crosslinking increasing the viscosity of the sample when the temperature rises. After the degree of crosslinking further increases, the fluidity of the sample decreases, and the bonding effect decreases. Therefore, 140 °C is thought of as the appropriate crosslinking temperature of DCP to EVA resin.

**Figure 3:**
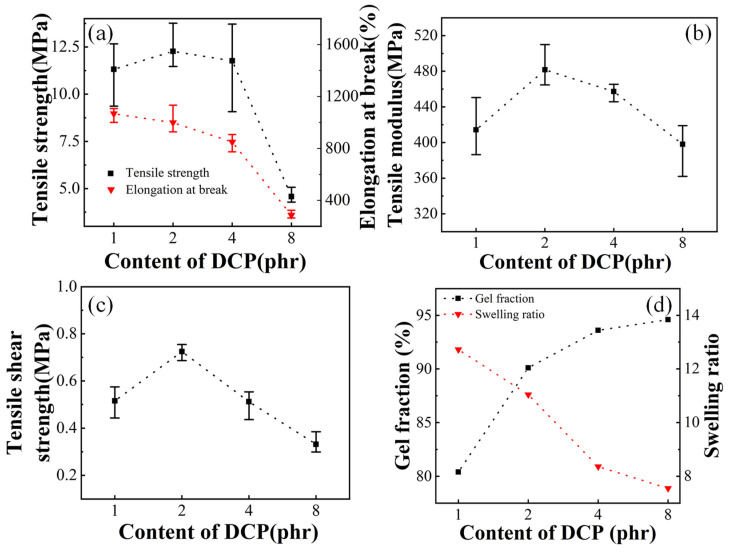
Tensile strength, elongation at break (**a**), tensile modulus (**b**), tensile shear strength (**c**), gel fraction and swelling ratio (**d**) of Elvax220w samples with different DCP content.

The degree of crosslinking is also an important factor affecting the mechanical properties of EVA hot melt adhesive, so the influence of DCP content needs to be considered. The tensile strength, elongation at break, tensile modulus, tensile shear strength, gel fraction and swelling ratio for vulcanized Elvax220w samples by different DCP content are shown in Figure 3. It can be seen from Figure 3d that with the increase of DCP content, the gel content gradually increases, and the swelling degree gradually decreases, indicating that with the increase of DCP content, the crosslinking degree of the sample gradually increases. When the content of DCP is 1, 2 and 4 phr, the tensile strength of the vulcanized samples does not change much. When the content of DCP increases to 8 phr, the tensile strength of the sample decreases significantly. The elongation rate gradually decreases with the increase of DCP content. Tensile modulus increases first and reaches maximum at 4 phr and subsequently it begins to decrease with the increase of DCP content. The increase of the tensile strength and tensile modulus in the initial stage as the degree of crosslinking increases may be due to the fact that EVA resin without crosslinking is difficult to crystalize and its strength is very low. As the degree of crosslinking continues to increase, the tensile strength begins to increase, and generally reaches a maximum. If the crosslinking is excessive, the subsequent tensile strength will also decrease, and the elongation at break will also decrease. Finally, the crosslinking reaction will damage the EVA structure to a certain extent, thus breaking the molecular chain and reducing the strength.

Figure 3c shows the tensile shear strength of vulcanized Elvax220w samples with different DCP content. It can be seen that the tensile shear strength of EVA sample with 1 phr DCP has been significantly improved. When 2 phr DCP is added, the tensile shear strength further increases and reaches the maximum. With the further increase of DCP content to 4 phr, the tensile shear strength begins to decrease, which may be due to the fact that DCP content is too high so that the crosslinking process is too rapid, resulting in the decrease of the wetting effect between the EVA sample and PC sheets. When the DCP content increases to 8 phr, the decrease in tensile shear strength seems to be more obvious. Therefore, 2 phr DCP should be appropriate to effectively improve the bonding strength of the EVA adhesion.

### 3.3. Influence of Silane Coupling Agent

**Figure 4:**
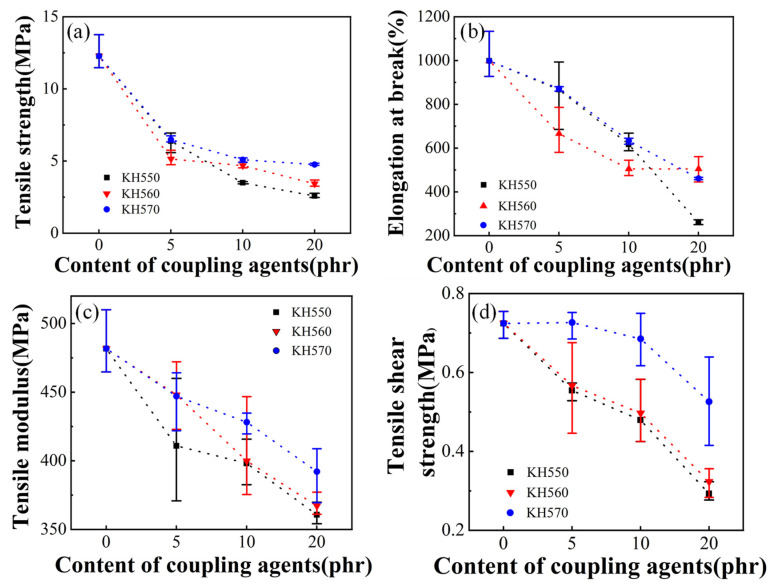
Tensile strength (**a**), elongation at break (**b**), tensile modulus (**c**) and tensile shear strength (**d**) of EVA/DCP samples with different silane coupling agent.

**Figure 5:**
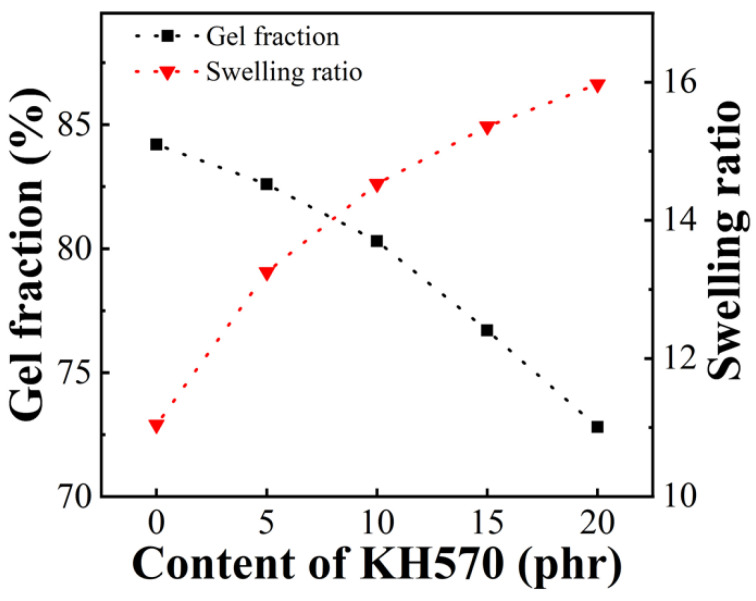
Gel fraction and swelling ratio of EVA/DCP samples with different KH570 content.

Three kinds of silane coupling agents KH550, KH560, KH570 were used to compound with EVA resin Elvax220w. Here, 2 phr of DCP is used. Figure 4a–c shows the tensile strength, the elongation at break and the tensile modulus of EVA sample with different silane coupling agent content, respectively. It can be seen that with introduction of the silane coupling agent, the tensile strength, the elongation at break and the tensile modulus decrease rapidly, and the decrease is the slowest when the KH570 is added. When the amount of silane coupling agent reaches 10 phr, the decreases in tensile strength and elongation at break begin to slow, or even almost unchanged, while the tensile modulus continues to decrease by a larger margin. It can be considered that the silane coupling agent preferentially reacts with DCP, which results in the slowing down of the crosslinking of EVA and the greatly reduced crosslinking degree, which can be seen from Figure 5. With the increase of KH570 content, the gel content gradually decreases, and the swelling degree gradually increases, indicating that with the increase of KH570 content, the crosslinking degree of the sample gradually decreases. The reactivity of KH550 with DCP is the highest, and the reactivity of KH570 with DCP is the lowest. The continuous decrease in tensile modulus is due to the addition of liquid in the system, which reduces the elasticity during stretching.

Figure 4d shows tensile shear strength of the EVA sample with different silane coupling agent content. It can be seen that the tensile shear strength of the samples with KH570 increases with the increase of KH570 content at first, and then there is an obvious decrease; for the samples with KH550 and KH560, the tensile shear strength decreases with the increase of the silane coupling agent content. This is due to the fact that the added KH550 and KH560 have high reaction activity with DCP, resulting in a low degree of crosslinking of EVA itself. The added KH570 has low reactivity with DCP, and the EVA resin still has a certain degree of crosslinking, which increases the viscosity of the system and enhances the bonding strength between EVA and PC sheets. In addition, the addition of KH570 can improve the fluidity of the system and make the adhesive have better wettability with PC sheets. KH570 has strong polarity after hydrolysis, which can also enhance the bonding effect of the adhesive to a certain extent.

**Figure 6:**
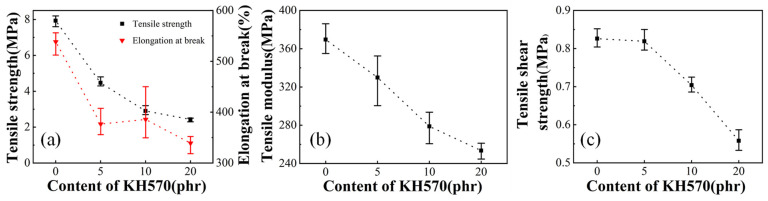
Tensile strength, elongation at break (**a**), tensile modulus (**b**) and tensile shear strength (**c**) of Elvax220w/EVA43/DCP/KH570 samples with different KH570 content.

In general, the effect of adding KH570 to the EVA system is better than those of KH550 and KH560. Among them, the introduction of 5 phr KH570 can increase the tensile shear strength to a certain extent, which is also the most important performance of the adhesive. After adding KH570, both the tensile strength and elongation at break ratio decreased, but it still has good performance. The influence of KH570 content on Elvax220w/EVA43 blends was investigated. As shown in the Figure 6, similar to the sample without EVA43, as the content of KH570 increased, the tensile strength, elongation at break and tensile modulus of the sample gradually decreased, which is due to the addition of liquid and the reduction of the degree of cross-linking between EVA and DCP. While unlike the samples without EVA43, as the content of KH570 increased, the tensile shear strength of the samples with EVA43 immediately began to decrease. Generally speaking, in this multi-blend system, EVA43 effectively improves the tensile shear strength of the adhesive, but it leads to a decrease in tensile performance. This is because the added EVA43 has more advantages than the Elvax220w matrix because of its high VA content and viscosity. The addition of an appropriate amount of DCP promotes the cross-linking of EVA, which improves the overall tensile properties and bonding properties of the system, but the improvement of DCP in the Elvax220w/EVA43/KH570 system is relatively not as obvious as in the Elvax220w/KH570 system. The addition of KH570 did not improve performance in this multicomponent system.

## 4. Conclusions

By comparing different EVA matrix resins, EVA blends of Elvax220w/EVA43 with appropriate viscosity and tensile shear strength were found to be more suitable as hot melt adhesives. The preparation and curing conditions of EVA compound hot melt adhesive and the effect of DCP on the properties of hot melt adhesive were studied. As a result, the optimum temperature and dosage of DCP were determined. When DCP content was 2 phr and 5 phr KH570 was added, the tensile shear strength was increased from 0.247 Mpa to 0.726 Mpa. At the same time, the obtained samples also had good tensile properties. Silicone coupling agents with different functional groups were added to EVA compound hot melt adhesive, but the results show that due to the consumption of DCP, the improvement of tensile strength and tensile shear strength of the hot melt adhesive is very small, and it was easier to cause performance degradation.

## Figures and Tables

**Table 1:** Melt index, VA content and molecular weights of EVA copolymers.

Code	Melt Index ^a^ (dg/min)	VA Content(wt%)	*M*_n_^b^ × 10^4^	*M*_W_^b^ × 10^4^	Tensile Shear Strength (MPa)
Elvax210w	400	28	1.73	4.36	0.207
Elvax220w	150	28	2.11	5.67	0.247
Elvax420	150	18	1.14	1.76	0.107
EVA43	43	32	-		0.885

^a^ Melt index by ASTM D1238. ^b^ Measured by GPC.

**Table 2:** Codes and structure of silane coupling agents.

Code	Chemical Name	Chemical Formula	Boiling Point (°C)
KH550	γ-aminopropyltriethoxysilane	NH_2_CH_2_CH_2_CH_2_Si(OC_2_H_5_)_3_	217
KH560	γ-(2,3-epoxypropoxy)propytrimethoxysilane	(C_2_H_3_O)CH_2_OCH_2_CH_2_CH_2_- Si(OC_2_H_5_)_3_	290
KH570	γ-Methacryloxypropyltrimethoxysilane	CH=C(CH_3_)COOCH_2_CH_2_CH_2_-Si(OCH_3_)_3_	255

## Data Availability

The data presented in this study are available on request from the corresponding author.

## Supplementary Materials

The following are available online at https://www.mdpi.com/article/10.3390/polym13234101/s1, Figure S1: Samples for testing tensile shear strength; Figure S2: DSC curves of Elvax220w and EVA43; Table S1: The half-life of DCP at different temperatures.
