# Supplementary material for: Effects of Crosslinking and Silicone Coupling Agent on Properties of EVA Composite Hot Melt Adhesive
Source: Polymers (Basel). 2021 Nov 25;13(23):4101. doi: 10.3390/polym13234101 (PMC8659007; doi:10.3390/polym13234101)
Supplement: Supplementary file 1 [file polymers-13-04101-s001.zip › polymers-1458997-supplementary.pdf]

Supporting Materials for

Effects of Crosslinking and Silicone Coupling Agent on Properties of EVA Composite Hot Melt Adhesive

Zijin Wu[a], Yonggang Shangguan[a, *], Chunhui Zhang[b], Qiang Zheng[a, *]

[a]*MOE Key Laboratory of Macromolecular Synthesis and Functionalization, Department of Polymer Science and Engineering, Zhejiang University, Hangzhou 310027, P. R. China*

[b]*Zhejiang Liniz New Material Co., Ltd, Hangzhou 311300, P. R. China*

The samples used for testing tensile shear strength were made according to Chinese national standard GB-T7124-2008 as following.

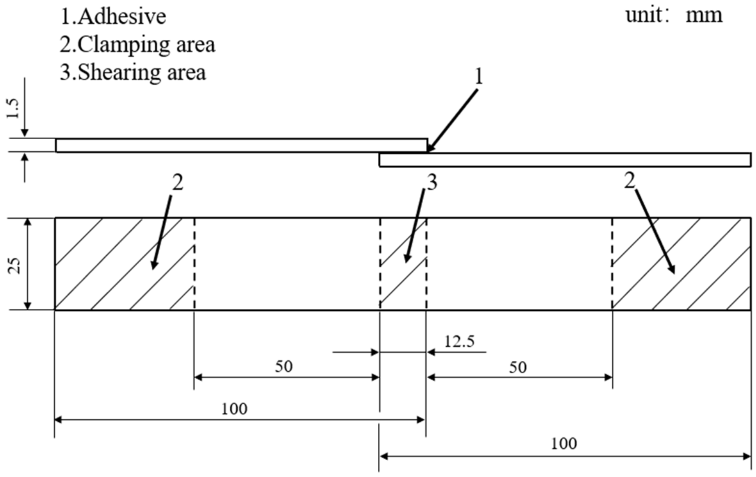

Figure S1. Samples for testing tensile shear strength.

DSC of Elvax220w and EVA43 were messured, and the results were shown as Figure S2.

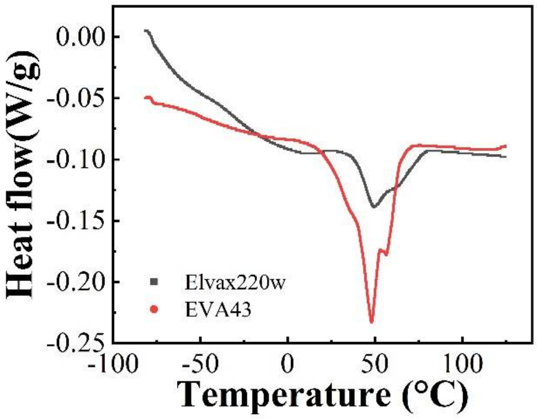

Figure S2. DSC curves of Elvax220w and EVA43.

Table S1. The half-life of DCP at different temperatures.

**Temperature（℃）**	**113–117**	**125**	**150**	**171**	**180**
Half-life（min）	6000	138	15	1	0.4
